# CT-guided special approaches of drainage for intraabdominal and pelvic abscesses: One single center's experience and review of literature

**DOI:** 10.1097/MD.0000000000012905

**Published:** 2018-10-19

**Authors:** Ning Zhao, Qian Li, Jing Cui, Zhiyong Yang, Tao Peng

**Affiliations:** a Department of Gastrointestinal Surgery; b Department of Radiology; c Department of Pancreatic Surgery, Union Hospital, Tongji Medical College, Huazhong University of Science and Technology, Wuhan, China.

**Keywords:** inaccessible abscess, multiplane reconstruction, percutaneous drainage, transintestine

## Abstract

**Background:**

To explore the safety and efficacy of several special approaches of drainage for deep inaccessible intraabdominal and pelvic abscesses.

**Methods:**

By searching of our institutional database, the clinical and radiologic information of all patients with special approaches of abscesses drainage was collected, consisting of etiology, diameter of abscess, duration of drainage, major complications, rates of success, failure and death, and pre-procedure, intra-procedure and post-procedure computed tomography scans.

**Results:**

A total of 124 patients are eligible for the criterion in our center between January 2010 and January 2018. The mean diameter of abscess was 5.6 cm (range 3.0–9.8 cm) and mean duration of drainage was 10.3 days (range 4–43 days). Pain was complained in 6 patients (4.8%) and hemorrhage was observed in one patient. Complete resolution of the abscess following drainage was observed in 115 patients (92.7%). A total of 9 patients (7.3%) failed to percutaneous abscess drainage and 3 patients died of catheter-unrelated diseases. Transintestinal afferent loop of drainage was firstly attempted in six patients and complete resolution of abscess was achieved in five patients.

**Conclusion:**

Special approaches, including transgluteal, presacral space, transhepatic, multiplane reconstruction (MPR)-assisted oblique approach and transintestinal afferent loop approach for those deep inaccessible intraabdominal and pelvic abscesses are safe and feasible.

## 1 Introduction

A variety of disorders, including perforated hollow viscera, inflammatory bowel disease, and postsurgical complications, might produce intraabdominal and pelvic abscesses, which could result in a series of complications, such as abdominal hemorrhage, prolonged hospitalization, and higher mortality.^[1]^ Owing to tremendous advances in diagnostic imaging and interventional radiology techniques over the past few decades, image-guided percutaneous drainage has been the standard therapy for intraabdominal and pelvic abscess.^[2,3]^ Conventional approaches of abscess drainage are sometimes inaccessible due to many obstacles, such as intestine, etc.

The purposes of this study are to explore the safety and efficacy of several special approaches of drainage for deep inaccessible intraabdominal and pelvic abscesses by combining our experience and previous reports.

## 2 Materials and methods

This study protocol was approved by the ethics committee of our college. Informed consent to perform special approaches of abscess drainage was obtained from each patient before the procedure.

By searching our institutional database, the clinical and radiologic information of all patients with special approaches of abscesses drainage was collected, consisting of etiology, diameter of abscess, duration of drainage, major complications, rates of success, failure and death, and pre-procedure, intraprocedure, and post-procedure computed tomography (CT) scans. For all patients, the indication of percutaneous abscess drainage was validated by the interventional radiologist and the physician. Abscesses were considered as inaccessible via conventional approaches, which was confirmed by 2 experienced interventional radiologists. Seldinger technique was preferred in our center because it is more controlled than the tandem-trocar technique. If clinical, laboratory, radiologic improvement was observed, and the amount of daily drainage was less than 10 mL/24 h, the catheter was removed.

Success is defined as appropriate placement of the drainage catheter in the abscess cavity and improved clinical symptoms. Failure is defined as the need for surgery or recurrence after the removal of catheter. Any cause of death was also considered failures. Catheter-related complications were classified as major complications (catheter-related pain, hemorrhage, infection, and visceral injury along the access route) and minor complications (catheter dislodgement, obstruction, and kinking) according to the Society of Interventional Radiology guidelines.^[4]^

## 3 Results

A total of 124 patients, including 57 men and 67 women with a mean age of 50.6 years (range 27–78 years), are eligible for the criterion in our center between January 2010 and January 2018, and their clinical information is demonstrated in Table 1. The mean diameter of abscess was 5.6 cm (range 3.0–9.8 cm) and mean duration of drainage was 10.3 days (range 4–43 days). Six patients (4.8%) complained of pain and no other major complications were noted. Complete resolution of the abscess following drainage was observed in 115 patients (92.7%). A total of 9 patients (7.3%) failed to percutaneous abscess drainage and 3 patients died of catheter-unrelated diseases.

**Table 1:**
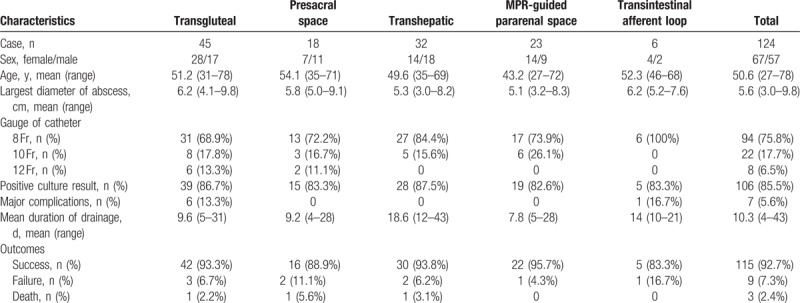
Characteristics of patients with special approach.

### 3.1 Transgluteal approach

**Figure 1:**
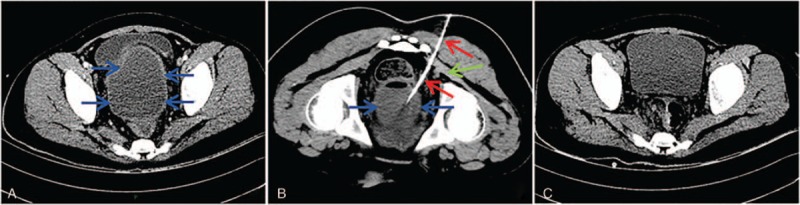
Transgluteal approach. (A) Axial CT scans revealed that 8 × 9 cm abscess (blue arrow) is detected in the excavation rectovesicalis. (B) Axial CT scans (prone position) showed a 17-gage coaxial needle (red arrow) advanced progressively into the abscess cavity (blue arrow) through the piriformis (green arrow). (C) Axial CT scans obtained 2 weeks after the removal of drainage catheter revealed that abscess cavity completely collapsed.

A total of 45 patients underwent CT-guided transgluteal pelvic abscess drainage and complete resolution of the abscess following drainage was observed in 42 patients (93.3%). The detailed characteristics of patients with transgluteal approach are summarized in Table 1. The fluid collections were related to postoperative complications in 33 patients (73.3%) and inflammatory or infectious intraabdominal disease in the remaining 12 patients (Appendicitis: n = 5; Acute diverticulitis: n = 6; Crohn disease: n = 1) (26.7%). The catheter advanced below the piriformis muscle (Infrapiriformis approach) in 34 patients (75.6%) and through the piriformis (Transpiriformis approach) in 11 cases (24.4%). Intraprocedural pain requiring the intravenous analgesics and postprocedural localized pain at the catheter site was observed in 2 cases (all with transpiriformis) and 4 cases (Infrapiriformis approach: n = 1; Transpiriformis approach: n = 3). No other major complications were noted. A total of 3 cases failed to transgluteal abscess drainage. Follow-up CT scan in 2 patients revealed that the abscess communicated with the bowel and subsequent operation was performed. One patient died of acute myocardial infarction 6 days after the placement of drainage catheter. The procedure of transgluteal approach is shown in Fig. 1.

### 3.2 Presacral space approach

**Figure 2:**
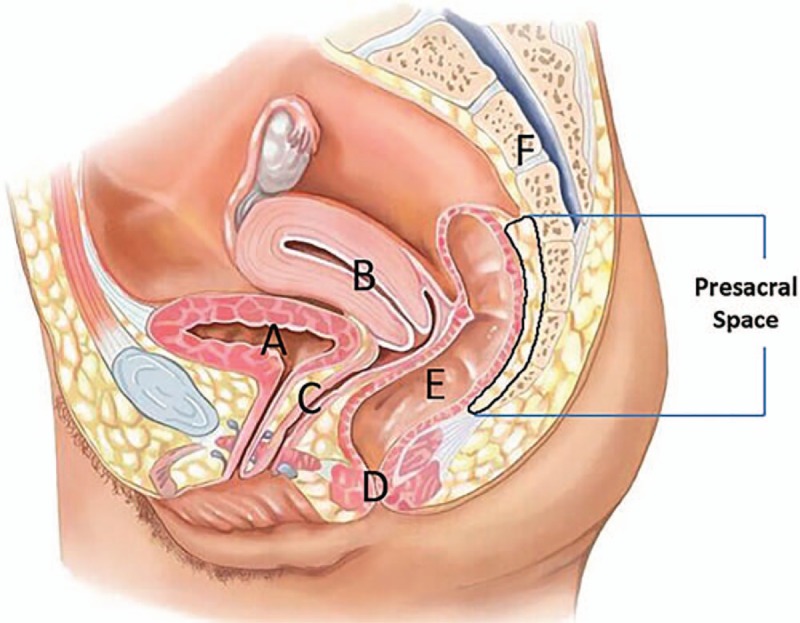
Drawing of female pelvis (midsagittal view) shows the anatomy of the presacral space. A = Urinary bladder, B = Uterus, C = Vagina, D = Anus, E = Rectum, F = Sacrum.

**Figure 3:**
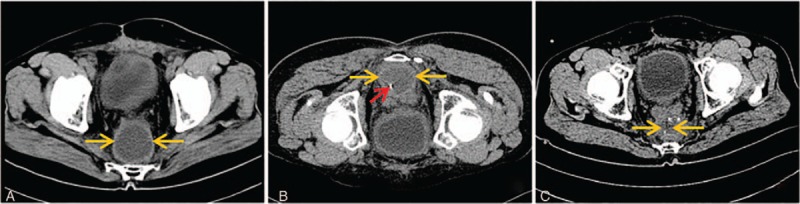
Presacral space approach. (A) CT scans revealed that 5 × 7 cm abscess (light orange arrow) is located between bladder and sacrum (the rectum had been resected). (B) When the patient was in prone position, intraprocedural CT scans demonstrated that the tip of needle (red arrow) advanced into presacral abscess (light orange arrow) during procedure. (C) CT scans obtained 3 days after the procedure revealed that abscess cavity (light orange arrow) obviously shrinked.

A total of 18 patients underwent CT-guided presacral space pelvic abscess drainage and complete resolution of the abscess following drainage was observed in 16 patients (88.9%). The detailed characteristics of patients with presacral space approach are summarized in Table 1. The fluid collections were related to postoperative complications in 15 patients (post-colorectal surgery: n = 11; post-peritonitis surgery: n = 4) (83.2%) and inflammatory or infectious intraabdominal disease in the remaining 3 patients (appendicitis: n = 1; acute diverticulitis: n = 1; Crohn disease: n = 1) (16.8%). The procedure was well tolerated in all patients (100%) and no major complications (such as hemorrhage or perforation) were noted. A total of 2 cases failed to presacral space abscess drainage. One patient with enteric fistula performed surgical abscess drainage and the other one died of pulmonary embolus 10 days after the procedure. The drawing of presacral space and the procedure of presacral space approach is shown in Figs. 2 and 3.

### 3.3 Transhepatic approach

**Figure 4:**
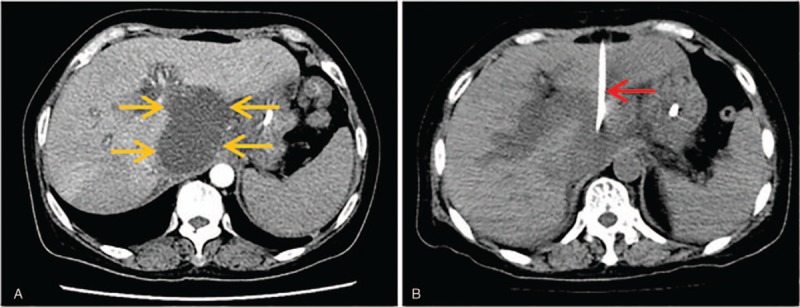
Transhepatic third segment approach. (A) Axial CT scans showed an abscess (light orange arrow) with size of 6 × 8 cm was detected in the lesser peritoneal sac. (B) Axial CT scans showed drainage catheter (red arrow) get through the third segment of liver parenchyma into the abscess cavity and abscess cavity completely collapsed ten days after the placement of drainage catheter.

**Figure 5:**
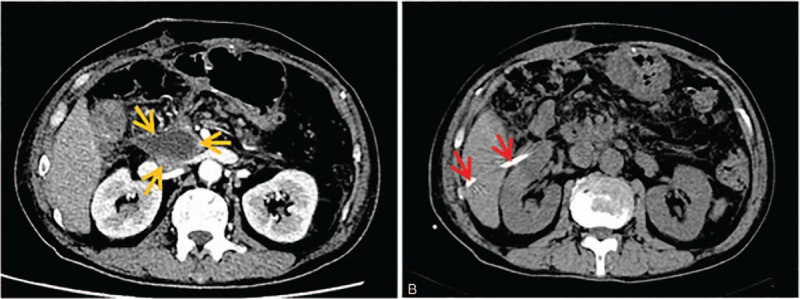
Transhepatic sixth segment approach. (A) Axial CT scans showed that an abscess (light orange arrow) with size of 3 × 4 cm was detected in previous region of pancreatic head and spread to the porta hepatis. (B) Axial CT scans showed drainage catheter (red arrow) get through the sixth segment of liver parenchyma into the abscess cavity and abscess cavity completely collapsed 12 days after the placement of drainage catheter.

A total of 32 patients underwent CT-guided transhepatic abscess drainage and complete resolution of the abscess following drainage was observed in 30 patients (93.8%). The detailed characteristics of patients with transhepatic approach are summarized in Table 1. The fluid collections were related to postoperative complications in 24 patients (gastric cancer: n = 6; pancreatic cancer: n = 12; bile duct cancer: n = 4; ampullary carcinoma: n = 2) (75%) and severe acute pancreatitis in 8 patients (25%). The access entrance was the left lobe of the liver in 17 cases (the third segment of liver parenchyma: n = 14) (53.1%) and the right lobe in the other 15 cases (the sixth segment of liver parenchyma: n = 12) (46.9%). No major complications (such as hemorrhage, bile leakage, secondary liver abscess, and bacteremia) were noted in all patients. A total of 2 cases failed to transhepatic abscess drainage. A recurrent peripancreatic abscess was observed in 1 patient with severe acute pancreatitis 2 months after the removal of catheter. Another patient with severe acute pancreatitis died of respiratory failure 8 days after the placement of drainage catheter. The procedure of transhepatic third and sixth segment abscess drainage is shown in Figs. 4 and 5.

### 3.4 Multiplane reconstruction (MPR)-assisted oblique approach

**Figure 6:**
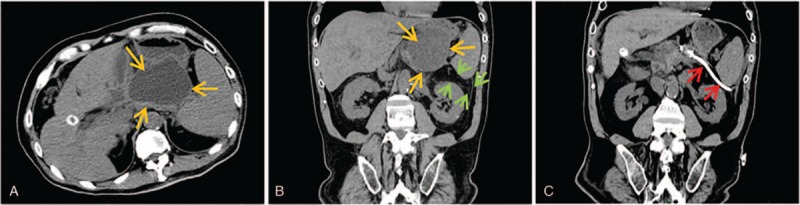
MPR-assisted pararenal space approach. (A) Axial CT scans showed an abscess (light orange arrow) with size of 7 × 8 cm was detected on top of pancreatic body and tail and surrounded by spleen and stomach. (B) MPR technique showed that a cross-sectional entrance along interstitial space of spleen and kidney (green arrow) into the abscess cavity (light orange arrow). (C) CT scans obtained 1 week after the procedure showed that drainage catheter (red arrow) advanced along the proposed access route and the abscess cavity collapsed.

A total of 23 patients underwent multiplane reconstruction (MPR)-assisted oblique abscess drainage and complete resolution of the abscess following drainage was observed in 22 patients (95.7%). The detailed characteristics of patients with MPR-assisted crossing pararenal space approach are summarized in Table 1. The fluid collections were related to postoperative complications in 15 patients (postgastric surgery: n = 11; post-pancreatic surgery: n = 4) (83.2%) and inflammatory or infectious intraabdominal disease in the remaining 3 patients (all with acute pancreatitis) (16.8%). One patient failed to MPR-assisted oblique abscess drainage due to enteric fistula. No death or major complications were noted in our study. The procedure of MPR-assisted oblique approach is shown in Fig. 6.

### 3.5 Transintestinal afferent loop approach

**Figure 7:**
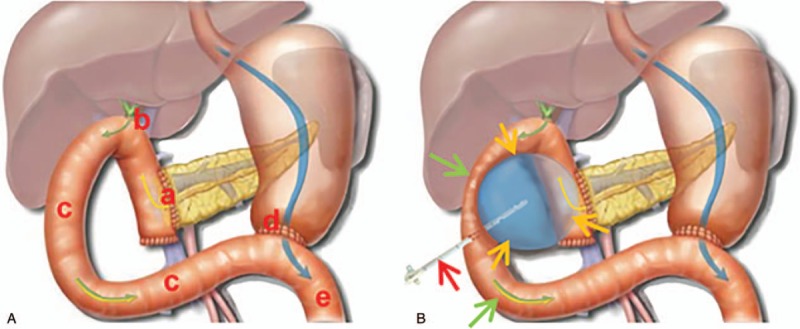
**(**A) Drawing of pancreaticoduodenectomy anatomy (coronal view): a: anastomosis of pancreas and intestine; b: anastomosis of bile duct and intestine; c: afferent intestinal loop; d: anastomosis of stomach and intestine; e: efferent intestinal loop. (B) Drawing of transintestinal afferent loop approach (coronal view): light orange arrow: abscess; green arrow: afferent intestinal loop; red arrow: drainage catheter.

**Figure 8:**
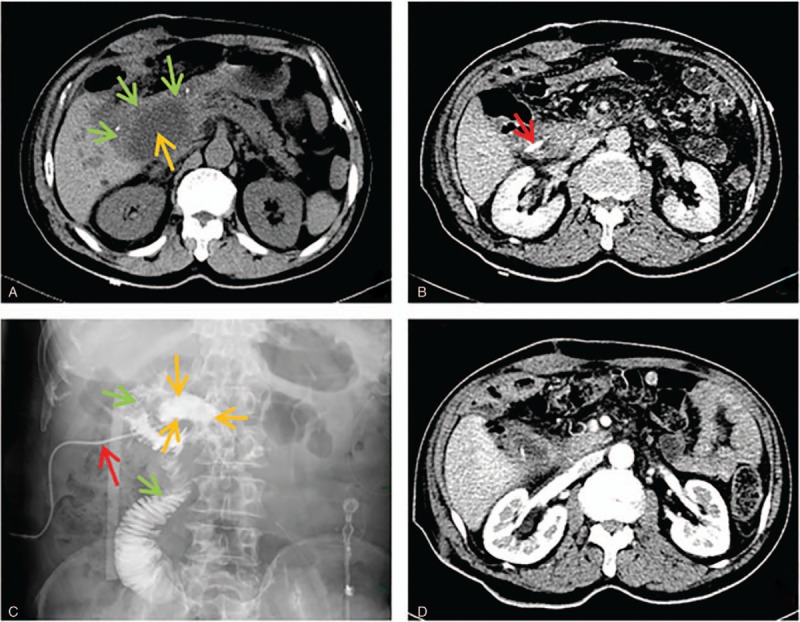
Transintestinal afferent loop approach. (A) Axial CT scans showed that an abscess (light orange arrow) with size of 5 × 6 cm was detected in previous region of pancreatic head and was surrounded by intestinal afferent loop (green arrow). (B) Axial CT scans obtained 3 days after the placement of drainage catheter showed that the tip of catheter (red arrow) was inside abscess cavity. (C) Drainage catheter pyrography obtained 1 week after the procedure confirmed that the drainage catheter (red arrow) got through the jejunum (green arrow) into the abscess cavity (light orange arrow). (D) Axial CT scans 10 days after removal of drainage catheter showed that abscess cavity completely collapsed.

A total of 6 patients underwent transintestinal afferent loop abscess drainage and complete resolution of the abscess following drainage was observed in 5 patients (83.3%). The detailed characteristics of patients are summarized in Table 1. Reoperation was needed in 1 patient due to the injury of mesenteric artery by puncture. No death was reported in our study. The drawing of pancreaticoduodenectomy anatomy and the procedure of transintestinal afferent loop approach are shown in Figs. 7 and 8.

## 4 Discussion

Special approaches for deep pelvic fluid collections drainage consist of transrectal, transvaginal, transgluteal, and presacral space approach. Undeniably, every approach has its inherent advantages and disadvantages. Although ultrasound-guided transvaginal or transrectal approach has advantages of real-time guidance and convenience, surrounding intestine or gas-containing abscess cavity often obscure the target and impede correct drainage catheter placement.^[5,6]^ Moreover, the potential risks of perforation of rectal wall and vaginal fornix, and retrograde infection of reproductive tract exist. If a long-term drainage is necessary, catheter fixation and patient discomfort would be rather tough for these access routes.^[7,8]^ CT could provide better contrast and spatial resolution for accurate localization of the collection and the detection of adjacent nervous and vascular structures.^[9]^ Therefore, CT-guided transgluteal drainage has been increasingly performed. Since the first report on transgluteal approach by Butch et al^[10]^ in 1986, few studies^[10–13]^ (Table 2) on the safety and efficacy of this procedure were reported. More than 95% of patients with transgluteal abscess drainage were reported to achieve complete resolution of abscess, whereas catheter-related intraprocedural and postprocedural pain was significantly associated with the access route, especially transpiriformis approach.^[11,13]^ Our observations are in agreement with their reports concerning the complete resolution of abscess and major complications of transgluteal deep pelvic abscess drainage. Furthermore, hemorrhage, gluteal abscess, and limitations in ambulation were occasionally detected in some reports.^[11,14]^ Compared with special approaches mentioned above, the most significant advantage of presacral space abscess drainage is well tolerance. Moreover, drainage catheter was placed parallel to the sacrum, which reduces disturbance to both bed rest and movement of lower extremities.^[15]^ However, enough presacral space induced by operation or inflammatory diseases is a necessary precondition for this access route.

**Table 2:**
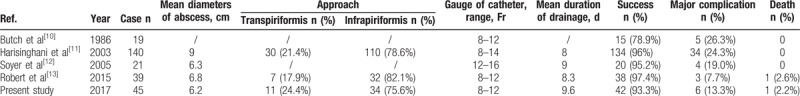
Literature on transgluteal approach of intraabdominal abscesses.

Since the initial report on transhepatic approach by Mueller et al^[16]^ in 1985, few reports^[16–18]^ (Table 3) have depicted the safety and efficacy of transhepatic approach. In 2009, Yamakado et al^[17]^ reviewed that 12 patients with real-time CT fluoroscopic-guided transhepatic drainage all achieved complete resolution of the abscess following drainage. In 2011, Ciftci et al^[18]^ reviewed 30 patients with transhepatic approach and got the similar result with complete resolution of abscess in 97% of patients. No major catheter-related complications (such as hemorrhage, bile leakage, secondary liver abscess, and bacteremia) were noted. Our observations are in agreement with their reports concerning the complete resolution of abscess and incidence of major complications of transhepatic intraabdominal abscess drainage. Moreover, owning to the absence of major vascular and biliary branches, the third and sixth segment of liver parenchyma is recommended as the optimal and prior transhepatic access route in our study.

**Table 3:**
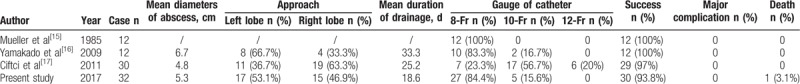
Literature on transhepatic approach of intraabdominal abscesses.

For procedures using CT-guided percutaneous abscesses drainage, oblique puncture is sometimes necessary to avoid injury of important organs and vessels. With the rapid development of CT post-processing technology, MPR technique of multislice CT is breaking the limitation of conventional axial plane and can be used as a tool for achieving oblique approach.^[19,20]^ The angio-CT system software could use an algorithm for super-rapid MPR to maintain the target position. Because it reconstructs 4 images (axial, sagittal, coronal, and oblique) and keeps the reference point with the previous image, we can see continuous images automatically without adjusting the position. Percutaneous drainage for subphrenic abscess and those abscesses in lesser peritoneal sac is usually challenging, because the transpleural approach can cause pulmonary complications, such as empyema and pneumothorax. Therefore, MPR is very useful in avoiding the transpleural approach.

Traversing intestine is usually forbidden in the process of percutaneous intraabdominal and pelvic abscess drainage, mainly worrying intestinal perforation. The first transintestinal afferent loop approach succeeded in an unexpected way. A 61-year-old female patient, with a surgical history of pancreaticoduodenectomy 2 months ago, presented with severe sepsis (hyperpyrexia and elevated serum inflammatory markers). Abdominal CT scans revealed that an abscess with size of 5 × 6 cm in previous region of pancreatic head squashed the intestinal afferent loop and had an obscure boundary. An 8-Fr catheter was unintentionally advanced through the intestinal afferent loop into the abscess cavity. Nevertheless, drainage catheter pyrography 1 week after the procedure revealed that jejunum was traversed. Frequent follow-up CT revealed that abscess completely collapsed and no clinical signs of intestinal perforation were noted. Finally, the catheter was successfully removed and patient's clinical presentation got markedly improved. Enlightened by the first successful case, we intentionally attempted the transintestinal afferent loop in 5 patients who underwent Billroth-II reconstruction and suffered from abdominal abscess surrounded by the intestinal afferent loop. Of the 5 patients, 4 patients achieved complete resolution of abscess and did not have clinical presentations of intestinal perforation; only 1 failed because of the injury of mesenteric artery by puncture. Our success was attributed to the following reasons. First, the gauge of 8-Fr catheter is appropriate in both draining abscess and minimizing intestinal damage. Second, gradually retracting and clipping drainage catheter gains enough time for the formation of sinus and the healing of intestinal break. Simultaneously, severe intraabdominal adhesion induced by operations could accelerate the process. Third, the fluid content (bile and pancreatic juice) travelling through afferent intestine loop has little possibility of obstructing drainage catheter. Therefore, it is presumed that the transintestinal afferent loop approach may be a feasible and safe access route for those abscesses secondary to Billroth-II reconstruction and surrounded by the intestinal afferent loop. However, more evidences are required to testify the hypothesis.

This study's limitations deserve commentary. First, due to the lack of definite practical guidance for inaccessible intraabdominal and pelvic abscesses, the approaches were made empirically instead of evidence-based. Second, this was a retrospective observational study from a single center, and the sample size of 2 original approaches (MPR-assisted oblique approach and transintestinal afferent loop approach) is relatively smaller. Therefore, the safety and efficacy of these approaches needs to be confirmed by large-sample, multicenter, randomized controlled trials.

## Author contributions

Ning Zhao and Tao Peng made contributions to the study design. Ning Zhao, Qian Li, and Jing Cui completed the literature search, data collection and analysis, and the initial manuscript. Zhiyong Yang and Tao Peng reviewed and revised the final manuscript.

**Conceptualization:** Ning Zhao, Tao Peng.

**Data curation:** Qian Li, Jing Cui.

**Formal analysis:** Ning Zhao, Qian Li.

**Methodology:** Qian Li, Jing Cui, Zhiyong Yang, Tao Peng.

**Resources:** Jing Cui.

**Software:** Ning Zhao, Tao Peng.

**Supervision:** Zhiyong Yang, Tao Peng.

**Validation:** Ning Zhao, Jing Cui, Tao Peng.

**Writing – original draft:** Ning Zhao.

**Writing – review & editing:** Zhiyong Yang, Tao Peng.

Tao Peng orcid: 0000-0003-1336-1772.
